# Myosin Va Brain-Specific Mutation Alters Mouse Behavior and Disrupts Hippocampal Synapses

**DOI:** 10.1523/ENEURO.0284-20.2020

**Published:** 2020-12-15

**Authors:** Swarna Pandian, Jian-Ping Zhao, Yasunobu Murata, Fernando J. Bustos, Cansu Tunca, Ramiro D. Almeida, Martha Constantine-Paton

**Affiliations:** 1 Department of Brain and Cognitive Sciences, McGovern Institute for Brain Research, Massachusetts Institute of Technology, Cambridge, MA 02139; 2 Institute of Biomedical Sciences, Faculty of Medicine and Faculty of Life Sciences, Universidad Andres Bello, Santiago, Chile 8370071; 3 Centre for Neuroscience and Cell Biology, University of Coimbra, Coimbra 3004-517, Portugal; 4 Instituto Superior Técnico Av. Rovisco Pais 1, 1049-001 Lisboa, Portugal

**Keywords:** anxiety, autism spectrum disorder, LTD, transport

## Abstract

Myosin Va (MyoVa) is a plus-end filamentous-actin motor protein that is highly and broadly expressed in the vertebrate body, including in the nervous system. In excitatory neurons, MyoVa transports cargo toward the tip of the dendritic spine, where the postsynaptic density (PSD) is formed and maintained. MyoVa mutations in humans cause neurologic dysfunction, intellectual disability, hypomelanation, and death in infancy or childhood. Here, we characterize the Flailer (Flr) mutant mouse, which is homozygous for a *myo5a* mutation that drives high levels of mutant MyoVa (Flr protein) specifically in the CNS. Flr protein functions as a dominant-negative MyoVa, sequestering cargo and blocking its transport to the PSD. Flr mice have early seizures and mild ataxia but mature and breed normally. Flr mice display several abnormal behaviors known to be associated with brain regions that show high expression of Flr protein. Flr mice are defective in the transport of synaptic components to the PSD and in mGluR-dependent long-term depression (LTD) and have a reduced number of mature dendritic spines. The synaptic and behavioral abnormalities of Flr mice result in anxiety and memory deficits similar to that of other mouse mutants with obsessive-compulsive disorder and autism spectrum disorder (ASD). Because of the dominant-negative nature of the Flr protein, the Flr mouse offers a powerful system for the analysis of how the disruption of synaptic transport and lack of LTD can alter synaptic function, development and wiring of the brain and result in symptoms that characterize many neuropsychiatric disorders.

## Significance Statement

Here, we characterize a mutant mouse homozygous for a Myosin Va (MyoVa) mutation named Flailer (Flr). The Flr mutation generates a dominant-negative MyoVa transport motor protein that sequesters synaptic cargo and blocks synaptic transport, thereby resulting in an absence of long-term depression (LTD) and in abnormal behaviors similar to those seen in anxiety and autism spectrum disorders (ASDs). We propose that the Flr mutant can be used as a model to study how the absence of LTD disrupts brain connectivity and behavior. Moreover, by using the Flr mutation together with gene editing technologies it should be possible to target specific brain areas to remove the mutation and recover MyoVa function, thereby interrogating the role of a specific brain region in the control of a particular behavior.

## Introduction

Myosin Va (MyoVa) is a plus-end filamentous-actin motor protein that is highly expressed throughout the vertebrate body, including in the nervous system (Mercer et al., 1991; Hammer and Sellers, 2011). Mutant MyoVa in humans produces an early lethal disease called Elejalde or Griscelli syndrome Type I, which is characterized by abnormal pigmentation and severe neurologic symptoms (Griscelli et al., 1978; Elejalde et al., 1979; Pastural et al., 1997; Durán-McKinster et al., 1999; Sanal et al., 2000). Similar to humans with Griscelli syndrome, MyoVa null mice (*dilute-lethal*) die early, either *in utero* or before postnatal day (P)21. However, rodent strains with small spontaneous *myo5a* mutations display milder phenotypes and have made possible studies of abnormal cerebellar function (Takagishi and Murata, 2006; Miyata et al., 2011).

The Flailer (Flr) mouse is homozygous for a *myo5a* mutation caused by a spontaneous non-homologous recombination event that produced a fusion gene lacking the coding region for the actin-binding “feet” of the MyoVa protein. The actin-binding domains are critical, since they allow MyoVa to “walk” toward the positive end of, and move between, actin filaments (Hammer and Sellers, 2011; Wagner et al., 2011). In the Flr mutant, this *myoVa* gene is fused to the promoter of the gene guanine nucleotide-binding protein subunit β-5 (*gnb5*) and as a consequence is expressed extensively in the CNS (Watson et al., 1994; Zhang et al., 2000, 2011; Witherow and Slepak, 2003; Lein et al., 2007; *Allen Brain Atlas*). When the Flr mutant protein is in a 1:1 ratio with wild-type (WT) *myo5a* protein, the Flr protein competes with WT MyoVa for cargo by forming abnormal myosin dimers that sequester cargo but are unable to step toward the postsynaptic density (PSD) on actin, thereby diminishing MyoVa function. In the WT brain, MyoVa carries many critical molecules and complexes along actin filaments in dendritic spine necks to distal spine synapses (Jones et al., 2000; Hammer and Sellers, 2011), including vesicle-bound molecules such as receptors, mRNAs translated at the synapse (Fujii et al., 2005; Yoshimura et al., 2006), and synaptic scaffolding complexes (Gerrow et al., 2006; Moutin et al., 2012; Yoshii et al., 2013). These scaffolding complexes are necessary to localize receptors and organize other molecules at the relatively isolated spine tip PSDs. Interestingly, the Flr phenotype is expressed only when the *flr* mutant gene is present in at least as many copies as the WT *myo5a* gene. More specifically, the Flr phenotype requires two copies of the *flr* mutant gene (hence appears to be recessive, although there is no second “WT” allele of the *flr* gene) in a WT *myo5a* background with two WT *myo5a* alleles, but requires only one copy (hence appears to be dominant) in a *myo5a* heterozygote with one *myo5a* null allele and one WT *myo5a* allele. These observations suggest that the *flr* mutation acts as a *myo5a* dominant-negative mutation (Jones et al., 2000).

Researchers studying MyoVa function have designed similar truncated constructs and over-expressed them in a variety of cells *in vitro* in attempts to block WT MyoVa function; however the high and scattered expression levels make the interpretation of these data difficult (Correia et al., 2008). Conversely, Flr animals show intact cellular function and behavior that is essentially the same in every animal and thus provide an excellent model organism for the study of MyoVa function in the brain.

Previously we characterized some of the synaptic defects of the Flr mouse visual cortex (VC), where we found long-term potentiation (LTP) to be normal but no evidence of long-term depression (LTD; Yoshii et al., 2013). Moreover, AMPA receptor (AMPAR) endocytosis was defective, AMPAR miniature current frequencies were significantly increased, and AMPAR to NMDA receptor (NMDAR)-evoked current ratios were abnormally high in VC neurons as recorded in brain slices. Also, immunocytochemical studies of cultured VC pyramidal cells showed generally increased expression of AMPARs, which in Flr mice are located in dendritic shafts rather than in spines because of the diminished transport by MyoVa (Yoshii et al., 2013).

Here, we characterize the Flr animal using behavioral testing, biochemistry and electrophysiology. These animals show repetitive grooming, anxiety, asocial behaviors, defective hippocampal memory, and abnormal pup vocalizations. The behaviors suggest that the Flr protein disruption of glutamate synapses produces an anxiety, obsessive compulsive-like phenotype similar to those seen in mouse strains in which neuropsychiatric candidate genes have been mutated (Welch et al., 2007; McFarlane et al., 2008; Peñagarikano et al., 2011; Peça and Feng, 2012). In addition, we observed a severe deficit in the transport of synaptic components to the synapse and a lack of LTD in the hippocampus of Flr animals. Our study highlights the important role for LTD in the etiology of mouse and presumably human, autism spectrum disorder (ASD)-related syndromes and, if combined with gene editing technologies, offers a new experimental approach to identifying the brain circuits responsible for neuropsychiatric disorders.

## Material and Methods

### Animals

All animal manipulations were performed following the guidelines of the Massachusetts Institute of Technology Institutional Animal Care and Use Committee (IACUC). Flr mice, line B6CBACa-A*^WJ^*/A, *tb^2J^/tb^2J^* F_2_ from the frozen embryo repository at Jackson Laboratory (Bar Harbor, ME), were bred as described in Jones et al. (2000) and supplied to us as pairs from their BcC-flr congenic line by the laboratory of Professor Miriam Meisler, University of Michigan, Ann Arbor, MI. Animals were rederived and maintained in the MIT IACUC. BcC-flr mice used for the current experiments were crossed with Thy-1 EGFP mice (Feng et al., 2000) to obtain F1 heterozygotes and subsequently bred to homozygosity for the *flr* gene. WT_FLR_-GFP animals were also obtained from these crosses. Both strains were bred for a subsequent four years in our laboratory. Genotypes were determined by PCR from mouse ear clippings (http://www.transnetyx.com/Services/Automated Genotyping. aspx). Animals were maintained at 23°C in the MIT IACUC facility with a 12/12 h light/dark cycle and food and water available *ad libitum*.

### Quantitative Western blotting

Synaptosomes were prepared as previously reported in (Yoshii et al., 2013). Briefly, hippocampal tissues were collected from P25 to P29 WT or Flr animals of both sexes in ice-cold TEVP buffer containing the following: 10 mm Tris-HCl, 5 mm NaF, 1 mm Na_3_VO_4_, 1 mm EDTA, 1 mm EGTA, pH 7.4, and 320 mm sucrose. Samples were homogenized using a tissue grinder and centrifuged at 1000 × *g* to remove nuclei and large debris. Supernatants were centrifuged at 10,000 × *g* and the pellet was resuspended in TEVP buffer. Pellets were centrifuged at 25,000 × *g* to collect the synaptosomal membrane fraction. After each centrifugation pellets was rinsed with ice-cold TEVP buffer to reduce contamination among fractions. Concentrations were determined with BCA assays (Pierce). Proteins were electrophoretically separated on 4–12% SDS-PAGE gels and transferred to a PVDF membrane (Millipore). Membranes were subsequently blocked with blocking buffer (Sigma) and then incubated with primary antibodies overnight at 4°C, rinsed, and then incubated with secondary antibodies for 30 min at room temperature (RT). Primary antibodies used were: rabbit anti-MyoVa (Sigma, LE18), mouse anti-TLS (Santa Cruz, sc-47 711), rabbit anti-mGluR1 (Millipore, 07-617), rabbit anti-mGluR5 (Neuromics, RA16100), mouse anti-fragile X mental retardation protein (FMRP; Millipore, 1C3), mouse anti-PSD-95 (NeuroMab, K28/43), mouse anti-PSD-93 (NeuroMab, N18/30), mouse anti-SAP102 (NeuroMab, N19/2), mouse anti-SAPAP1 (NeuroMab, N238/29), mouse anti-Shank1 (NeuroMab, N22/21), mouse anti-Shank3 (NeuroMab, N367/62), mouse anti-GluN1 (NeuroMab, N308-48), rabbit anti-GluN2A (Millipore, A12W), mouse anti-GluN2B (NeuroMab N59/20 and N59/36), mouse anti-GluA2 (NeuroMab, L21/32), mouse anti-homer 1 (NeuroMab, L113/130), mouse anti-IP3R (NeuroMab, N18/30), rabbit anti-tubulin β III (Abcam, ab6046), and mouse anti-actin (Sigma, AC-74). Blots were developed using chemiluminescence (Pierce. IL). Tubulin-βIII or β-actin bands on the same blot were used as loading controls. Band intensities, were confirmed to be in the linear range and then quantified using ImageJ software. At least three separate protein samples were used for quantification.

### *In vitro* electrophysiology

Acute slices were prepared from either Flr or WT_FLR_. Animals were anesthetized with isoflurane and decapitated. Brains were quickly removed and chilled in ice-cold dissection buffer. Transverse dorsal hippocampal 350- to 400-μm sections were cut using a VT-1000S vibratome (Leica) in ice-cold carbogenated sucrose solution containing the following: 240 mm sucrose, 2.5 mm KCl, 1 mm CaCl_2_, 5 mm MgSO_4_, 26 mm Na_2_HCO_3_, 1 mm NaH_2_PO_3_, and 11 mm glucose, transferred to a chamber containing aerated artificial CSF (ACSF) for 30 min at 32°C then maintained at RT (22–24°C) for a minimum of 1 h before electrophysiology recordings. Electrodes from borosilicate glass (WPI Sarasota) were pulled to 1- to 3-MΩ tip resistance using a P-97 puller (Sutter Instruments). For recordings, slices were submerged and perfused (3 ml/min) in a carbogen-saturated ACSF solution at RT. Stimulating electrodes were fabricated from tungsten bipolar electrodes (WPI) driven by ISO-STIM-01D (NPI Electronic GmbH). Field potentials were recorded in stratum radiatum of CA1, amplified with a MultiClamp 700B Axopatch 200B, digitized with a Digidata 1440A, filtered at 2 kHz, sampled at 10 kHz and stored in a computer that provided both on–line display and off-line data analysis using pClamp 10.2 and Clampfit 10.2 software (Mol. Devices Corp.).

### Dendritic protrusion density analysis

Male WT and Flr GFP mice were anesthetized with isoflurane and transcardially perfused with PBS followed by 4% paraformaldehyde (PFA) in PBS. Brains were dissected and postfixed with 4% PFA at 4°C for at least 12 h. The hippocampus was removed for transverse sectioning. Images were obtained with a confocal microscope (Nikon PCM2000 MVI) using a 60× oil-immersion objective with 2× magnification. Multiple 0.5-μm optical sections were used to analyze dendritic protrusions using Neurolucida Software (MBF Bioscience).

### Behavior analysis

Adult male mice were used for all behavior analyses except for pup vocalizations. All observers were blind to the phenotype of the mouse unless otherwise stated.

### Grooming behaviors

After 10 min of habituation to a fresh cage, mice were videotaped for 24 h under 700 lux day lighting (12 h) and ∼2 lux red light at night (12 h) illuminations. Grooming behaviors recorded at the onset of dark cycle (7–8 P.M.) were studied using an automated home-cage behavioral phenotyping system (Jhuang et al., 2010) designed by the Poggio laboratory in the McGovern Institute with manual corrections. The videos shown were taken in Plexiglas cubicles to illustrate details in Flr grooming.

### Elevated plus maze

We used a standard elevated plus maze as described previously (Munekazu et al., 2008). Each test session lasted 5 min. Scoring was accomplished using Ethovision XT Noldus Observer software.

### Three-chamber social approach test

The three-chamber test for sociability was performed as previously described (Moy et al., 2004) with minor modifications. A WT_FLR_ mouse was placed in the central chamber separated by opaque panels with holes providing passage between chambers. A novel male mouse was held in a wired cage allowing visual, tactile, and olfactory contact. Video recordings of the introduced mouse were taken for the following 10-min period.

### Social proximity tests

Social proximity testing was conducted in a clear rectangular chamber constructed of acrylic plastic under dim red light as previously described (Defensor et al., 2011). For testing, two non-cage male mice were simultaneously placed in the testing chamber for a 10 min trial. Either two mice of the same strain or one Flr and one WT_FLR_ were placed in the chamber. The entire test period was videotaped from two cameras providing front and side views. All behaviors involved some contact between the two animals.

### Morris water maze

Before the spatial memory trials, mice were taught the location of a transparent lucite platform (10 × 10 cm) submerged just underneath the surface of the water. Two sets of trials were performed from four independent drop location (East, West, South, and North of the platform) on training day. Memory for the platform location was assessed during four consecutive spatial trials 24 h later. For all spatial trials, swim distance (meters) and swim speed (meters per second) were recorded.

### Fear conditioning

Standard fear conditioning paradigms were used to test for freezing to context in standard fear-conditioning chambers housed in sound-attenuating boxes (MED Associates, St. Albans, VT). Twenty-four hours after a 5-min placement in the chamber with a 180-s noise (85-dB) stimulus followed immediately by a 2-s (0.6-mA) mild shock, mice were placed back into the same chambers. Freezing to the context provided by the box alone was assessed over 5 min. Cued stimulus fear conditioning was evaluated 2 h later in altered chambers. Freezing in the absence of the cued stimulus was assessed during the first 180 s of the test session, then the noise (85 dB) was presented and freezing to this cued stimulus was assessed.

### Communication deficits

Pups (P2–P14) were isolated from their mother and littermates for 10 min. For testing, pups were gently placed into a glass isolation container containing clean bedding material and surrounded by a sound attenuating box (18 × 18 × 18 cm) made of 4-mm-thick Styrofoam. An ultrasound Gate system monitored ultrasonic vocalization (USV) emission. Acoustic data were recorded with a sampling rate of 250,000 Hz in 16-bit format by a recorder (version 2.97; Avisoft Bioacoustics). For acoustical analysis, recordings were transferred to Avisoft SASLab Pro (version 4.50; Avisoft Bioacoustics)

### Data analysis

Statistical analyses were performed using GraphPad Prism software. *t* test paired, unpaired *t* test or ANOVA tests were performed as described in each figure; *p ≤ 0.05, **p ≤ 0.001, ***p ≤ 0.0001.

## Results

### Flr animals show anxiety, memory defects, and deficits in social interactions

The *flr* gene consists of the promoter and first two exons of brain-specific *gnb5* plus part of the adjacent intron fused in-frame with an intron of *myo5a*, allowing the *gnb5* promoter to drive the expression of truncated *myo5a* and express the Flr protein (Jones et al., 2000) at levels equal to WT MyoVa in the many brain regions in which Gnb5 is expressed (Zhang et al., 2000). Gnb5 forms complexes with members of the regulators of G-protein signaling (RGS) family to control G-proteins, including mGluR6 (Rao et al., 2007). In response to light and photoreceptor glutamate release, mGluR6 activation closes a non-selective cation channel in ON-retinal bipolar cell dendrites (Morgans et al., 2007). This closure depolarizes the otherwise hyperpolarized cells.

Because abnormal expression of the Flr protein in retinal bipolar cells might seriously compromise vision, we first tested the grating acuity of four mature (P56–P84) Flr and four age-matched WT mice with the Flr genetic background, hereafter referred to as WT_FLR_. We used scoptopic conditions, the virtual oculomotor system designed and constructed by Prusky et al. (2004). WT_FLR_ animals showed average grating acuities in their right and left eyes of 0.388 and 0.389 cycles per degree (cyc/deg), respectively, while the Flr mice showed a somewhat lower acuity of 0.345 cyc/deg for both eyes, indicating that despite a slightly lower acuity the Flr mutants were sufficiently visual to detect their environments and have reasonably normal environmental stimulation of their central nervous systems.

**Figure 1.**
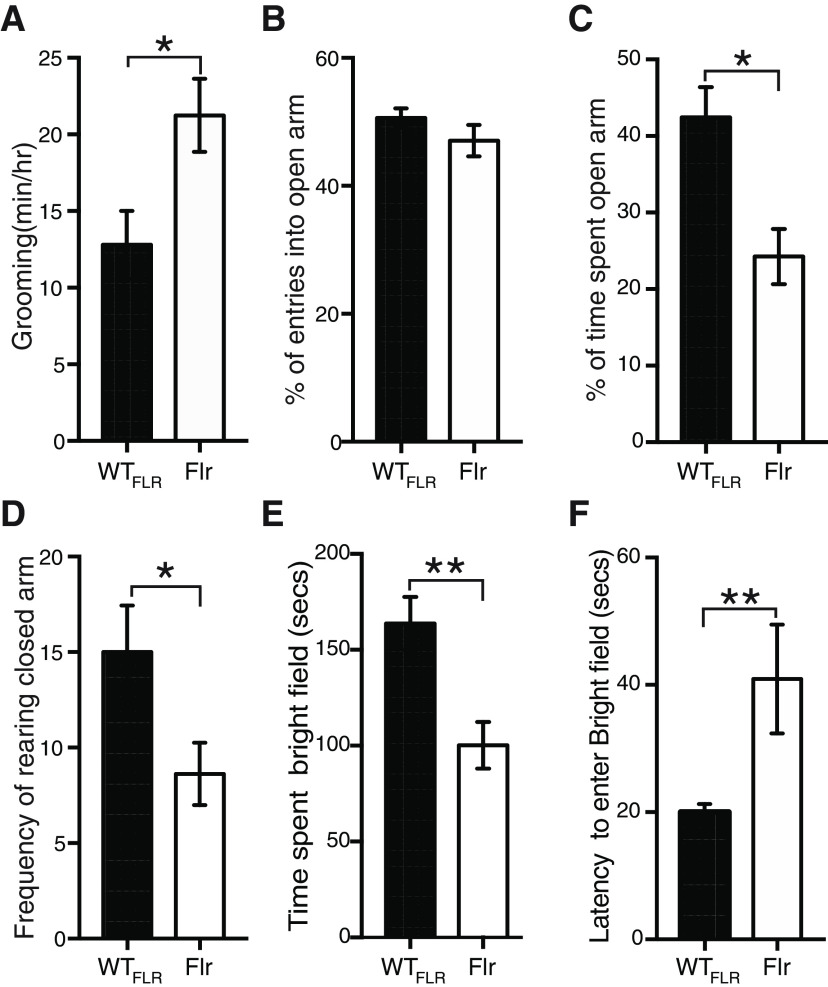
Flr mice show anxiety-like behaviors. ***A***, Quantification of the time spent grooming (*n* = 6 for both Flr and WT_FLR_; Movies 1, 2). ***B–D***, Elevated plus maze to test entries into the open arms (***B***), time spent in the open arms compared (***C***), and rearing (***D***). ***E***, Light and dark test to measure time spent in bright field (***E***) and latency to enter bright field (***F***; *n* = 11 for both WT_FLR_ and Flr). Unpaired *t* test was used for statistical analysis; *p ≤ 0.05, **p ≤ 0.001. Error bars represent ±SEM.

Next, using adult (P56–P84) male Flr and WT mice, we characterized the behaviors displayed by the animals. In all cases both training and testing took place within the first 3 h of the dark cycle. All behavioral tests were conducted using male animals, hence, our data are only valid for males. To determine the anxiety levels shown by Flr animals, we performed three tests. The most salient feature of Flr behavior as compared with that of the WT_FLR_ strain was the frequency and completeness of their spontaneous bouts of self-grooming (Fig. 1*A*; Movies 1, 2). Individual bouts could last up to 6 min and the sequence of this grooming behavior in Flr was nearly identical to the normal syntactical behavior described by Berridge and Whishaw (1992) for rats. This behavior is regularly associated with high anxiety in mice. Because this behavior involved full-body grooming by Flr, it did not cause the loss of head-fur as has been described for several mice strains with SAPAP or Shank3 deletions (Welch et al., 2007; Peça et al., 2011). In addition, we made use of the elevated plus-maze (McNaughton et al., 2008) and found no significant difference in the entries into the open arms between Flr and WT_FLR_ mice (Fig. 1*B*). However, Flr mice spent significantly less time in the open arms compared with WT mice, suggesting increased anxiety (Fig. 1*C*). Rearing in the closed arms, considered an exploratory behavior, was also significantly decreased in Flr versus WT_FLR_ (Fig. 1*D*), but frequencies of head dips in the open arm, considered a risk-taking behavior (McFarlane et al., 2008; McNaughton et al., 2008), were similar in Flr and WT_FLR_ (data not shown). Lastly, we tested the animals in a light/dark paradigm (Costall et al., 1989). We found that Flr animals spent less time in the bright field (Fig. 1*E*) and showed a higher latency to enter the dark field (Fig. 1*F*) than WT_FLR_ animals. Together, our results demonstrate that Flr animals show strong anxiety and repetitive behaviors.

Movie 1. Recording of grooming behavior of WT_FLR_ animal. Recording was performed in the dark cycle for 1 h in a plexiglass cubicle to illustrate details. 10.1523/ENEURO.0284-20.2020.video.1

Movie 2. Recording of grooming behavior Flr animal. Recording was performed in the dark cycle for 1 h in a plexiglass cubicle to illustrate details. 10.1523/ENEURO.0284-20.2020.video.2

**Figure 2.**
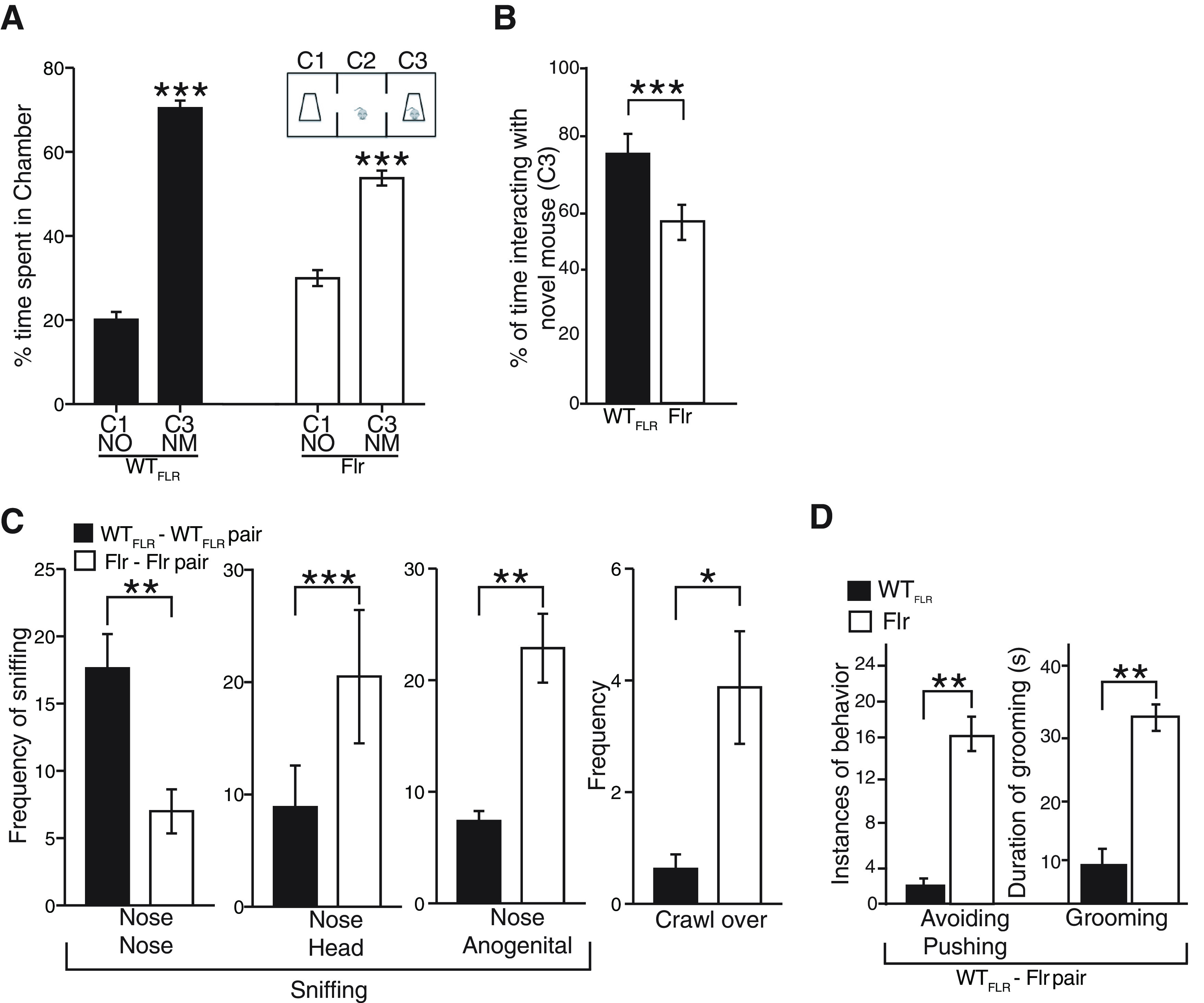
Atypical social interactions in Flr mice. ***A***, Three-chamber social approach test where a caged NM in chamber 3 (C3) and a NO in chamber 1 (C1) were placed (*n* = 17 for both WT_FLR_ and Flr). ***B***, Percentage of time spent interacting with NM (sniffing and close proximity to the NM cage; Movies 3, 4). ***C***, Social proximity test with Flr-Flr and WT_FLR_-WT_FLR_ pairs. The behaviors measured were nose tip-to-nose tip sniffing (with/without contact; NN), NH sniffing, NA sniffing (*n* = 20 for both WT_FLR_ and Flr). ***D***, Social proximity test with Flr-WT_FLR_ pairs Flr to quantify avoiding/pushing and grooming behaviors (Movies 5, 6). Unpaired *t* test and sum-rank test was used for statistical analysis; *p ≤ 0.05, **p ≤ 0.001, ***p ≤ 0.0001. Error bars represent ±SEM.

Another characteristic behavior of ASD phenotypes is the lack of aptitude to perform social interactions. To determine Flr’s ability to interact socially we used the three-chamber social interaction test (Bolivar et al., 2007; McFarlane et al., 2008; Defensor et al., 2011). In the three-chamber social interaction test WT_FLR_ and Flr mice spent approximately the same amount of time with the novel mouse (NM) and less but insignificantly different times in the chamber with the novel object (NO; Fig. 2*A*). Interestingly, when we reviewed the behavior of the mice in the chamber with the NM in detail and blinded to strain difference, we found that Flr spent significantly less time than the WT_FLR_ adjacent to the NM (Fig. 2*B*) and if adjacent to the NM, Flr spent most of its time grooming (Movies 3, 4). To gain insight into this behavior, we tested for direct interactions by quantifying social sniffing between animals. Flr/Flr or WT_FLR_/WT_FLR_ pairs were contained in a clear rectangular chamber (7 × 14 × 30 cm) that required some physical contact between the animals (Defensor et al., 2011). Flr pairs showed a significant reduction in nose-to-nose (NN) sniffing compared with WT_FLR_ pairs. There were also significant increases among Flr pairs compared with WT_FLR_ pairs in nose-to-anogenital (NA) sniffing and in nose-to-head (NH) sniffing. The latter might reflect attempts of Flr to avoid accidental nose-to-nose contact, a possibility consistent with the additional finding that crawl-over (CO) behavior by the recipient mouse, which avoids NN and NH sniffing, was also significantly increased in Flr pairs compared with WT_FLR_ pairs (Fig. 2*C*). To establish that general olfactory behavior was not disrupted in Flr, animals were tested in isolation for non-social sniffing behavior. Both WT_FLR_ and Flr spent similar amounts of time sniffing a water-saturated wick, and, in a separate exposure, a vanilla extract saturated wick, suggesting that general olfactory behavior of the strains was not perturbed (data not shown). Our data suggest that Flr/Flr pairs actively avoid head region close contact (Fig. 2*C*). To test this hypothesis, we placed Flr/WT_FLR_ pairs together in the same close proximity box, reasoning that if WT_FLR_ mice persisted in head region contact Flr might demonstrate a more obvious avoidance behavior. We found that the Flr member of each pair avoided the WT_FLR_ sniffing near the head by turning away abruptly, or actively, with forepaws pushing the WT_FLR_ away (Fig. 2*D*; Movies 5, 6). Moreover, Flr animals also engaged in significantly more instances of grooming-onset during this 2-min interval compared with WT_FLR_ (Fig. 2*D*), suggesting an increase in anxiety. Together, our data indicate that Flr animals display severe social deficits, avoiding repeatedly and strongly contact with other animals.

Movie 3. Recording of WT_FLR_ animal in the three-chamber social test. Animals were recorded for 10 min to determine social interaction. WT_FLR_ animals spend more time interacting with the novel animal than NO. 10.1523/ENEURO.0284-20.2020.video.3

Movie 4. Recording of Flr animal in the three-chamber social test. Animals were recorded for 10 min to determine social interaction. Flr animals spend more time close to novel animal than NO, but its time is spent grooming. 10.1523/ENEURO.0284-20.2020.video.4

Movie 5. Recording of Flr/WT_FLR_ pairs in a proximity box to demonstrate avoidance behaviors by Flr. When in close contact Flr animals turn their head abruptly to avoid contact. 10.1523/ENEURO.0284-20.2020.video.5

Movie 6. Recording of Flr/WT_FLR_ pairs in a proximity box to demonstrate avoidance behaviors by Flr. When in close contact Flr animals actively pushes WT_FLR_ animal with forepaws to avoid contact. 10.1523/ENEURO.0284-20.2020.video.6

**Figure 3.**
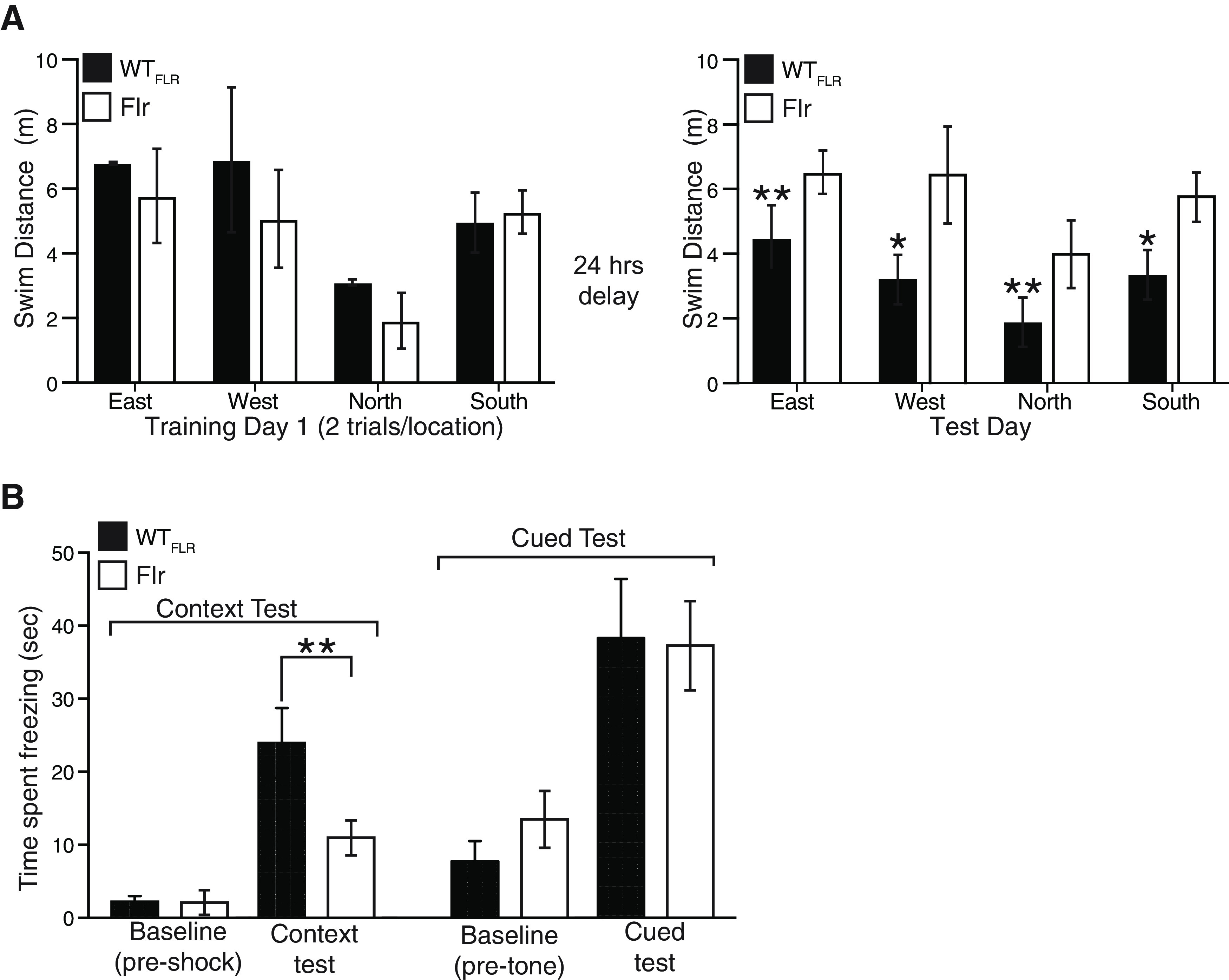
Flr mice show deficient hippocampus dependent-spatial memory formation. ***A***, Morris water maze on WT_FLR_ and Flr animals on training and test days (*n* = 9, 8 trials per animal on each training day). Animals were dropped in four different locations East, West, North, or South. After 24-h delay on test day, WT_FLR_ travels a significantly shorter distance to reach platform compared with training day. ***B***, Context-dependent and cued fear (85-dB noise) conditioning paradigms of WT_FLR_ and Flr animals. Unpaired *t* test was used to compare within a group between training and test day. One-way ANOVA was used to compare between groups. Error bars represent ±SEM; *p ≤ 0.05, **p ≤ 0.001.

To test whether memory formation in Flr animals is disrupted, we conducted the Morris water maze test and fear conditioning tests. For spatial memory each Flr or WT_FLR_ mouse was trained during repeated 60-s periods to find a submerged platform in an environment with distinct visual cues. Four different drop zones were used (North, South, East, West) and training was conducted until all mice found the submerged platform with similar efficiency (Fig. 3*A*, training day). Retention of the platform’s position was tested 24 h later. In this test, Flr mice failed to retain the memory of the platform position: they spent significantly less time in the quadrant containing the platform and covered significantly longer swim distances to reach the platform compared with WT_FLR_ (Fig. 3*A*, test day). Average swim speed (meters per second) was not significantly different between Flr mice and WT_FLR_ (Flr 0.1 m/s and WT_FLR_ 0.09 m/s). In addition, we tested memory formation dependent on either hippocampus or amygdala using the fear-conditioning paradigm based on context or cue, respectively. Each individual mouse was placed in a test box with a floor that could deliver a short electric shock (MED Associates). All mice explored the environment with little or no freezing. For hippocampal-dependent memory formation, in a second individual introduction to the box, every mouse received a 2-s, 0.6-mA electric shock to its feet (Fig. 3*B*, context test, baseline). When each mouse was subsequently re-introduced to the box, Flr mice showed a significant decrease in freezing compared with WT_FLR_ (Fig. 3*B*, context test), suggesting defects in hippocampal dependent learning/memory. To test for amygdala-dependent memory formation caused by fear, the apparatus consisted of the same box plus an 85 dB. burst of noise delivered before the shock. Both Flr and WT_FLR_ mice were first tested with a noise stimulus and a shock. Both strains of mice responded with freezing, and no significant difference was observed between Flr and WT_FLR_ (Fig. 3*B*, cued test, baseline). Animals were reintroduced to the box after ∼2 h and given the sound cue without the shock. Both Flr and WT_FLR_ spent approximately the same time freezing, indicating that cued-learning was not significantly different between WT_FLR_ and Flr (Fig. 3*B*, cued test). Together, our data indicate that Flr has severe hippocampal-dependent memory formation deficits and that fear-conditioned memory formation dependent on the amygdala is not affected.

**Figure 5.**
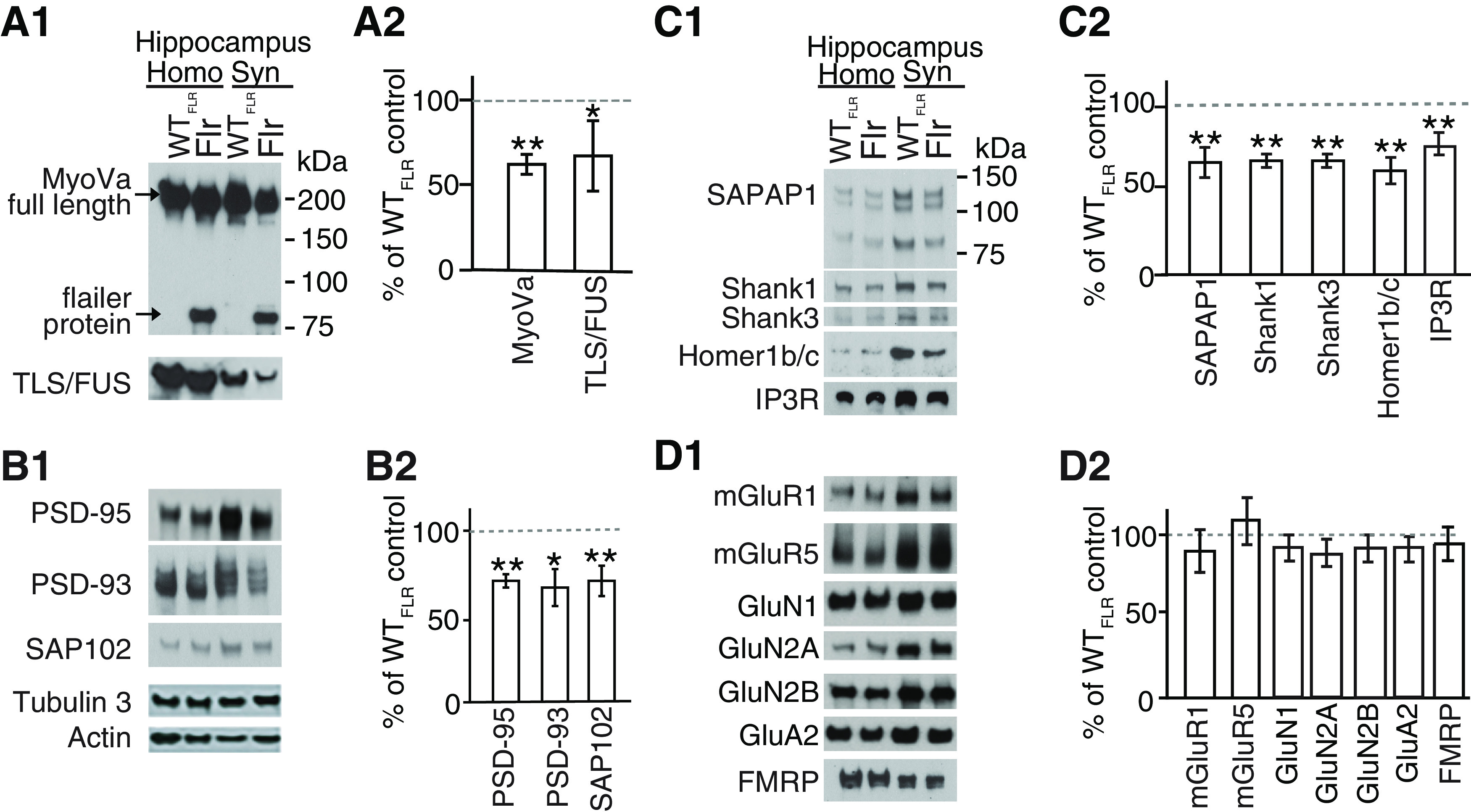
Synaptic transport of proteins to PSD is altered in Flr mice. WT_FLR_ and Flr hippocampal synaptosome extracts were used for quantitative Western blot analyses of synaptic proteins. ***A1***, ***A2***, Levels of full-length MyoVa and TLS/FUS were reduced in Flr extracts. ***B1***, ***B2***, Expression levels of major MAGUKs (PSD-95, PSD-93, SAP102) and (***C1***, ***C2***) SAPAP1, SHANK1, SHANK3, Homer1b/c, and IP3R were significantly reduced in Flr. ***D1***, ***D2***, mGluRs, NMDAR, AMPAR subunit levels, and FMRP were not changed in Flr when compared with WT_FLR_. Homogenate (Homo) and synaptosome (Syn) was prepared from whole hippocampus. Protein levels in Flr were quantified and plotted as % of WT_FLR_; *n* = 3 samples per group/each sample pool three to five animals. Unpaired *t* test was used for statistical analysis. Error bars represent ±SEM; *p ≤ 0.05, **p ≤ 0.001.

**Figure 4.**
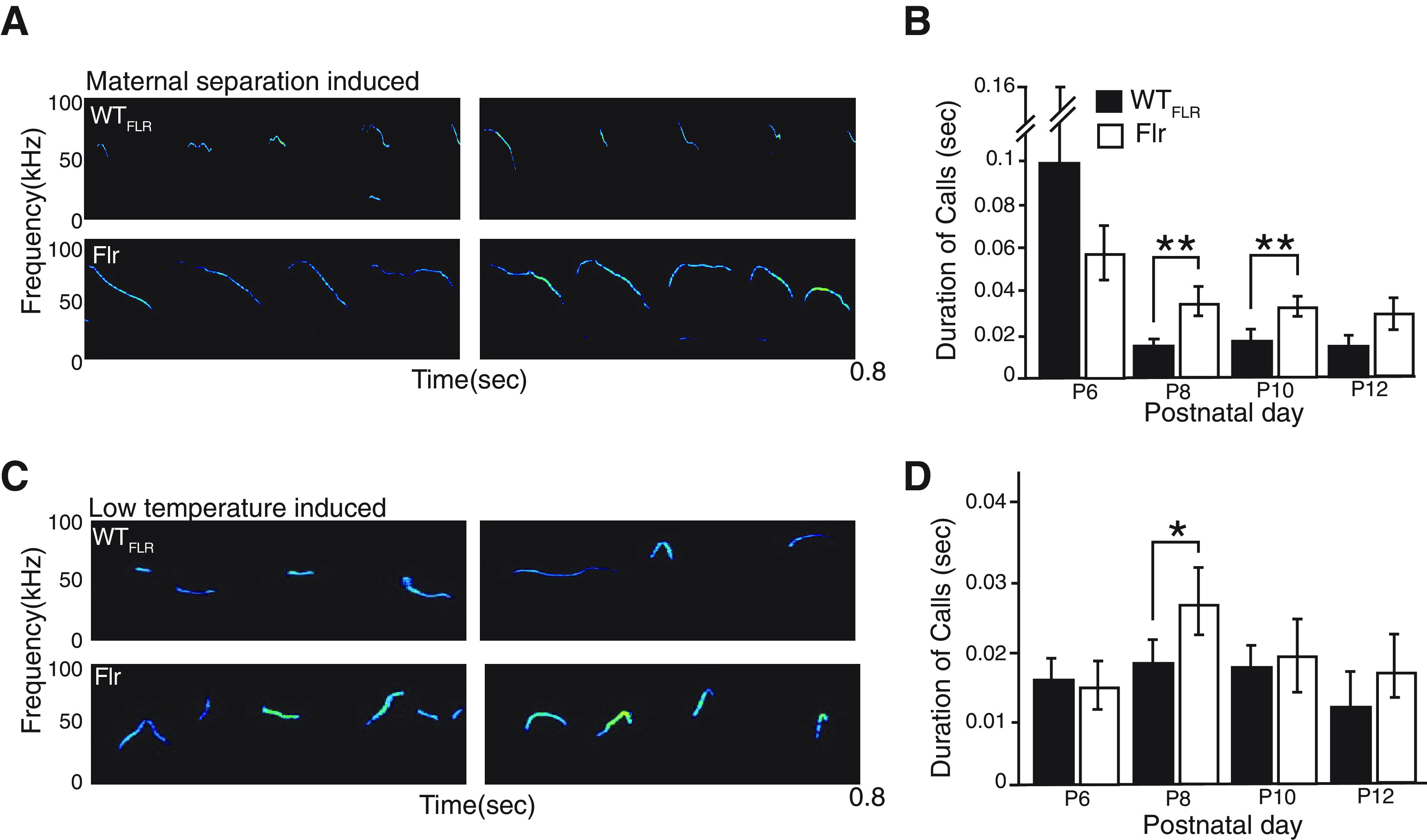
Communication deficits in Flr mice. ***A***, Representative spectrograms of a P8 pup during maternal separation. ***B***, Maternal isolation induced USV on P8 and P10 (*n* = 10 for both WT_FLR_ and Flr). Flr showed statistically significant increase in total duration of calls. ***C***, Representative spectrogram of P8 pup USV during cold-temperature-induced vocalization. ***D***, Cold temperature-induced USV (*n* = 10 for both WT_FLR_ and Flr). Flr showed statistically significant increase in total duration of calls on P8. Unpaired non-parametric *t* test was used for statistical analysis. Error bars represent ±SEM; *p ≤ 0.05, **p ≤ 0.001.

Another characteristic behavior observed for ASD mouse models is an increase in the duration of ultrasonic vocalizations (USVs) (Wöhr et al., 2011). This abnormal vocalization has been observed in BTBR T+tf/J and *Shank1* mutants, two models of ASD (Scattoni et al., 2008; Wöhr et al., 2011). To test whether Flr mice also display any defect in vocalization phenotype, we evaluated changes in USVs in Flr and WT_FLR_ pups after separating individuals from their mothers and littermates or after exposing isolated pups to low temperatures. Under separation conditions, all mouse pups produced infrequent and relatively long calls starting on P6. However, when the same pups were tested again at P8, and P10, isolated from their mothers and littermates, the duration of the Flr pup calls were invariably longer than those of WT_FLR_ (Fig. 4*A*,*B*). No changes in call frequency was observed. In addition, calls induced by low-temperature isolation also had longer durations in Flr than in WT_FLR_ but only at P8 (Fig. 4*C*,*D*). Taken together, our data show severe behavioral deficits of Flr animals, similar to what has been observed in mice with anxiety or ASD like phenotypes.

### Flr animals are deficient in the transport of synaptic components to the PSD

Next, we asked whether the severe changes in animal behavior observed in Flr are caused by the diminished transport of synaptic components to the PSD. The imaging analyses of Gerrow et al. (2006) and Moutin et al. (2012) demonstrated that glutamate synapse scaffolding molecules, SAPAP (GKAP), Shank and the membrane-associated guanylate kinase (MAGUK) PSD-95, travel as a complex up dendritic shafts and then up spine necks to the PSDs at the spine tip. In Flr, a significant amount of synaptic cargo gets transferred to a dimer of the truncated MyoVA cargo-binding domain (e.g., the Flr protein). These abnormal myosins, lacking the end-feet with ATP hydrolyzing heads of WT_FLR_ MyoVA, cannot step along the actin filaments to the PSD. However, it is possible than more than one MyoVa motor attaches to a single transport vesicle and moves in a straight line along an actin filament (Hammer and Sellers, 2011). This would account for the presence of Flr protein (∼85 kDa) in Flr mouse synaptosomes (Fig. 5*A1*). However, WT MyoVA carrying some of the scaffolds attached to the Flr protein cargo could be slowed down or unable to move to the PSD. This would account for the decrease in WT MyoVA found in Flr synaptosomes (Fig. 5*A1*,*A2*).

To characterize changes in transport of synaptic components in Flr neurons, we compared the levels of proteins between WT_FLR_ and Flr synaptosomes using quantitative Western blotting for 15 synaptic proteins. (1) Known MyoVA synapse-associated cargos: the mRNA binding protein TLS/FUS (Yoshimura et al., 2006), the IP3R (Takagishi et al., 1996; Miyata et al., 2011), and FMRP. (2) The major mature glutamate synapse scaffold complex, namely, PSD-95, SAPAP, and Shank (Gerrow et al., 2006; Moutin et al., 2012) and molecules closely related to PSD-95, namely, the MAGUKS PSD-93 and SAP102 (Nithianantharajah and Grant, 2013) and Homer1c known to be directly associated with Shank at synapses (Tu et al., 1999). (3) The major glutamate receptors or glutamate receptor subunits, namely, mGluR1, mGluR5, GluN1, GluN2A, GluN2B, and GluA2. Nine of these proteins TLS/FUS (Fig. 5*A1*,*A2*), PSD-95, PSD-93, SAP102 (Fig. 5*B1*,*B2*), SAPAP, Shank1, Shank3, Homer 1c, IP3R (Fig. 5*C1*,*C2*) showed significant reductions in their expression in Flr synaptosomes compared with synaptosomes from WT_FLR_ mice. However, all glutamate receptors and receptor subunits tested (mGluR1, mGluR5, GluN 1, 2A, 2B, A2) as well as the FMRP protein, were not significantly different in synaptosome levels between WT_FLR_ and Flr (Fig. 5*D1*,*D2*). Together, we found a significant reduction on the expression of the scaffolding complexes in synaptosomes of Flr neurons. Conversely, all receptors subunits studied did not show changes in the expression of proteins in Flr synaptosomes. This could be caused by the abnormal clustering of receptors at dendritic shafts (Yoshii et al., 2013) that might have rounded as vesicles and precipitated along with synaptosomes. The changes observed in synaptic composition because of the deficient transport by MyoVa could in fact explain the behavioral deficits observed in Flr mice shown above.

### mGLuR-dependent LTD, but not LTP, is impaired in Flr mutants

We have previously shown that greater than six-month-old Flr animals have impaired LTD in visual cortex slices, indeed showing a small LTP response in its place (Yoshii et al., 2013). Many studies have proposed that synaptic plasticity phenomena such as LTP and LTD are the main mechanisms that control memory formation (Nabavi et al., 2014). Here, we have shown that Flr animals have impaired hippocampal-dependent memory formation (Fig. 3), which could be a consequence of the lack of synaptic plasticity phenomena such as LTD. For this reason, we asked whether LTP and LTD at the Schaffer collateral (SC) to CA1 pyramidal synapse (SC-CA1) is altered in Flr mice.

**Figure 6.**
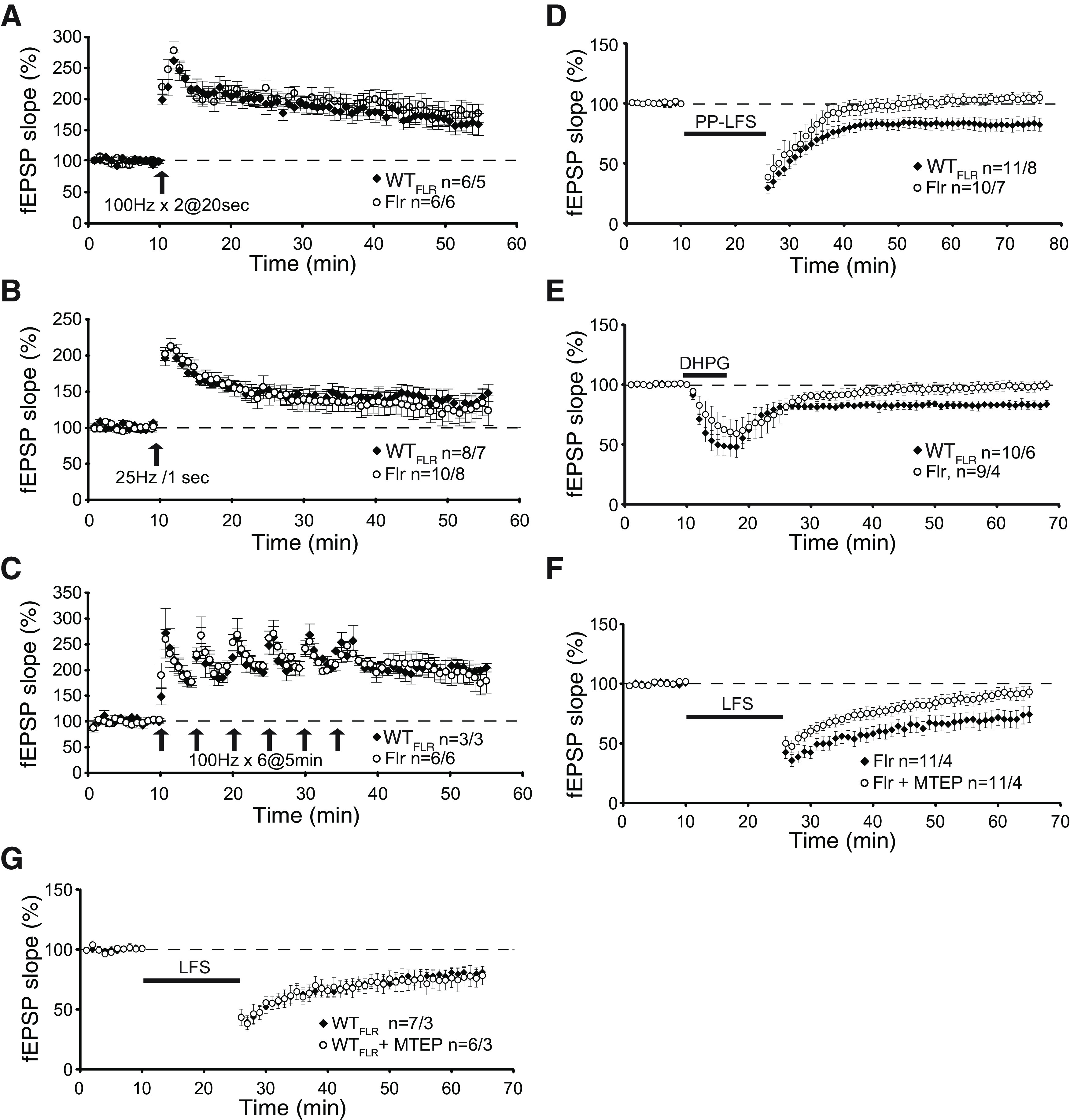
Absence of mGluR-dependent LTD at hippocampal SC to CA1 synapses in Flr mice. Field potentials (fEPSPs) were recorded in the stratum radiatum of CA1 by delivering stimulation to SC pathway in both WT_FLR_ and Flr mice. LTP was induced by (***A***) two 1-s 100-Hz bursts at 20-s interburst interval of 1-s duration and 20-s intertrain interval (arrow); (***B***) a single 1-s 25-Hz burst (arrow); (***C***) saturating induction, six 1-s 100-Hz bursts at 5-min interburst intervals (arrows). ***D***, mGluR-dependent LTD was induced In the presence of 50 μm D-AP5, PP-LFS (black bar; Kemp and Bashir, 1999). ***E***, Chemical induction of LTD using the mGluR agonist DHPG (50 μm) applied for 5 min (black bar). ***F***, Low-frequency stimulation (LFS) LTD (900 stimuli at 1 Hz, black bar) induced NMDAR-dependent LTD in Flr that is inhibited by MTEP (4 μm). ***G***, LFS of WT_FLR_ mice was not affected by the specific mGluR antagonist MTEP. n = number of slices/number of mice. Paired *t* test was used for statistical analysis. Error bars represent ±SEM.

To study fLTP in CA1 pyramids, we induced LTP by SC stimulation using three different protocols: two 1-s 100-Hz bursts at 20-s interburst intervals, involving both NMDARs and some L-type Ca^2+^ channels; a single 1-s 25-Hz burst known to be dependent only on NMDARs (Cavuş and Teyler, 1996); and saturation LTP, with six 100-Hz stimuli at 5-min intervals. In all three protocols used to induce LTP, responses between Flr and WT_FLR_ animals were indistinguishable, showing no defects in Flr animals (Fig. 6*A–C*, *n* = number of slices/number of mice). Conversely, two forms of LTD occur at SC-CA1 synapses. The first group requires mGluR5 activation of phospholipase C to generate IP3 and diacylglycerol (DAG). IP3 activates the IP3R on SER to release Ca^2+^ into spine cytoplasm and DAG triggers local protein synthesis (Huber et al., 2000). In WT rodents this form of LTD is induced by stimulating the SC-CA1 inputs with paired-pulses separated by 50 ms and given at 1 Hz in the presence of the NMDAR antagonist AP5 (Kemp and Bashir, 1999) or by application of the metabotropic Group1 mGluR agonist DHPG (Palmer et al., 1997). Both stimulation protocols produced normal mGluR5-dependent LTD in WT_FLR_ mice (Fig. 6*D*,*E*). In contrast, Flr mice showed a small LTP in response to the paired-pulse stimulation protocol and no LTD in response to the agonist DHPG (Fig. 6*D*,*E*). To determine the dependency on protein synthesis, we used the protein synthesis inhibitor anisomycin. In WT_FLR_ (control) mice mGluR5 LTD was blocked, showing a response similar the induction of LTP (*n* = 6 slices from 5 mice; data not shown). Conversely, Flr animals showed no mGluR5 LTD and a small LTP regardless of whether anisomycin was present (*n* = 15 slices from 13 mice without anisomycin; n= 5 slices from 5 mice with anisomycin; data not shown).

In contrast to mGluR5 LTD, NMDAR-dependent LTD induced by 900 stimuli at 1 Hz in Flr animals appeared normal (Fig. 6*F*, black squares), a finding also reported by Schnell and Nicoll (2001) based on recordings from young dilute-lethal mice. However, our data support the hypothesis that since some WT_FLR_ MyoVA could reach Flr synapses, there might be low, but sufficient, Ca^2+^ release through residual IP3R and mGluR5 expression/activity (Fig. 5) at the synapses of some cells to facilitate NMDAR-LTD. Consequently, we tested for an effect of residual mGluR5 facilitation of NMDAR-LTD by applying the highly specific mGluR5 antagonist MTEP (Cosford et al., 2003) to block any remaining mGluR5 activity in slices from Flr mice. We found that MTEP significantly reduced NMDAR-dependent LTD in these Flr brain slices (Fig. 6*F*, open circles). This effect was not observed in WT_FLR_ mice of the same age (Fig. 6*G*, overlapped data). This observation precludes any involvement in NMDAR-LTD of residual Ca^2+^ release from SER or tyrosine phosphorylation of NMDARs by Group1 mGluRs (Heidinger et al., 2002). Instead, these results indicate a mitigating effect of mGluR5 on the disruption of NMDAR-dependent LTD in Flr, resulting from the loss of normal scaffolding proteins.

**Figure 7.**
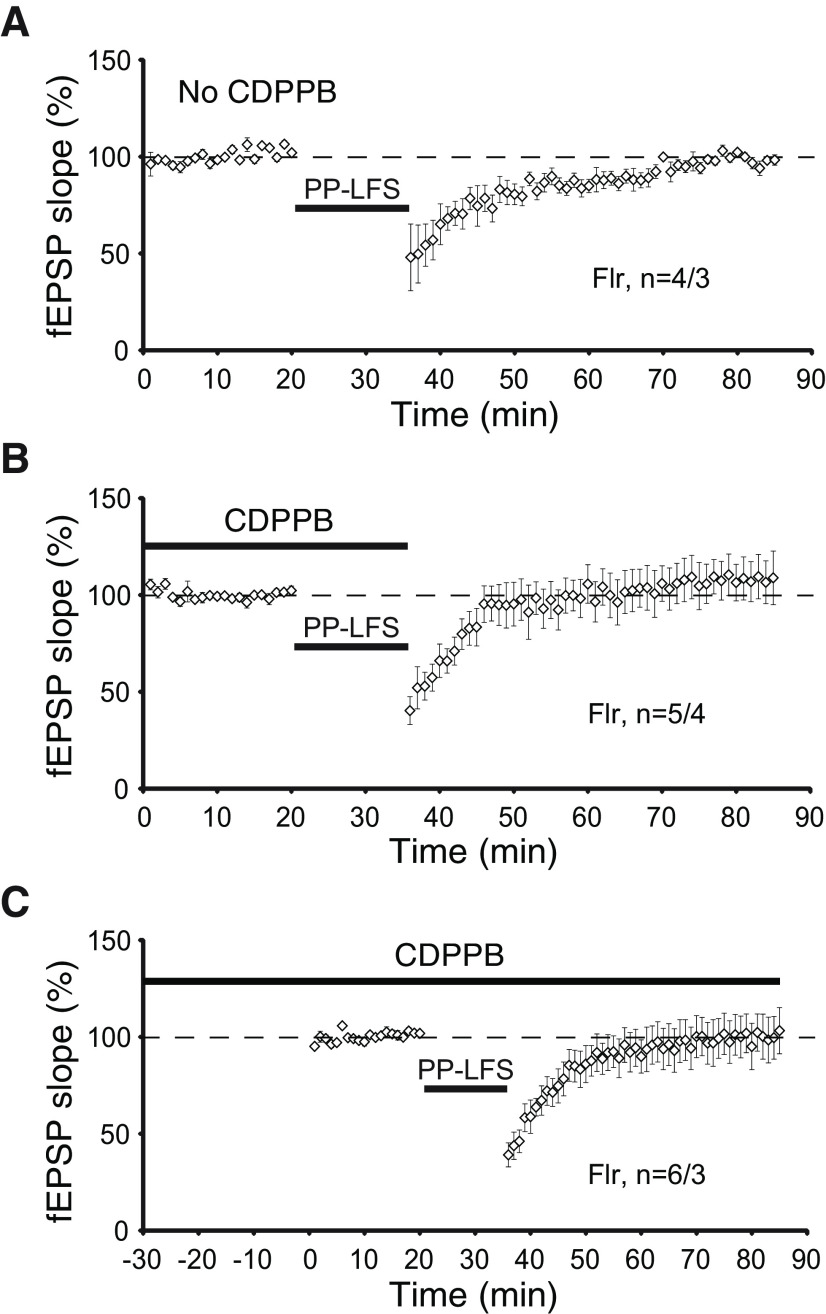
mGluR-dependent LTD is not rescued by mGluR5 agonist CDPPB in Flr mice. ***A***, LFS does not induce mGluR-dependent LTD in Flr animals. ***B***, CDPPB (10 μm) was added to the bath 30 min before and during LFS induction protocol for LTD. ***C***, CDPPB was added to the bath throughout the experiment, 30 min before, during, and after LFS mGluR-dependent LTD protocol. All recordings above were performed in the presence of 50 μm D-AP5. Paired *t* test was used for statistical analysis. Error bars represent ±SEM.

To confirm our conclusion that the loss of LTD is dependent on mGluR activity, we used a positive allosteric modulator (PAM) of mGluRs, 3-cyano-N-(1,3- diphenyl-1H-pyrazol-5-yl) benzamide (CDPPB; Kinney et al., 2005). In tuberous sclerosis Tsc2+/− mutant mice, a well-known intellectual disability, epilepsy and ASD animal model, the application CDPPB is able to restore normal mGluR-LTD (Auerbach et al., 2011). Consequently, we tested whether incubation of Flr hippocampal slices with CDPPB could rescue mGluR5-LTD defects. We found that pretreatment with CDPPB either applied to the slices for 35 min before recording (Fig. 7*B*) or applied for 30 min before and then during recording (Fig. 7*C*) did not restore defective mGluR-LTD in Flr (Fig. 7*A–C*). Together, our data show that in hippocampal slices Flr animals are able to produce normal LTP but that mGluR5-dependent LTD is severely impaired.

### Flr mutants have a reduced number of mature dendritic protrusions in CA1 pyramidal neurons

**Figure 8.**
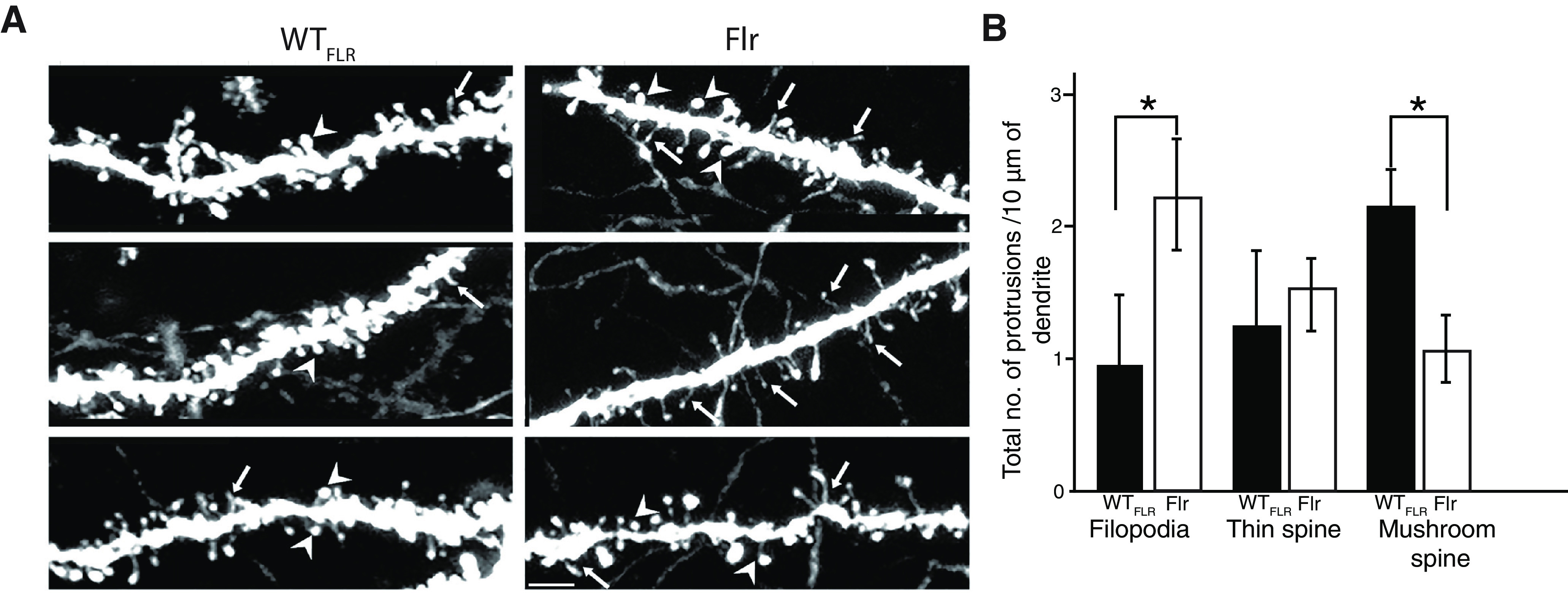
Flr shows increased number of filopodia and decrease in mushroom spines in apical dendrites of hippocampal pyramids. ***A***, Representative confocal images of apical dendritic shafts showing filopodia (arrows), thin spines (asterisk) and mushroom spines (arrowheads) in both Flr and WT_FLR_. ***B***, Quantification of the number of filopodia and mushroom spines in Flr and WT_FLR_ (*n* = 8 animals/genotype and 40 segments of length 15–20 μm). The spine analysis and reconstruction were done using autoneuron of neurolucida and contrast increase was done using ImageJ and adobe CC Scale bar: 10 μm. Unpaired *t* test with Welch’s correction was used for statistical analysis. Error bars represent ±SEM; *p ≤ 0.05.

In many human and mouse neurodevelopmental disorders, it has been shown an abnormal increase in spine density and filipodia shaped spines (Nimchinsky et al., 2001; Hutsler and Zhang, 2010; Peça et al., 2011; Penzes et al., 2011; Kulkarni and Firestein, 2012; Yoshii et al., 2013). To determine changes in spine morphology and density, we examined CA1 pyramidal cells, visible through expression of Thy1-GFP in this Flr strain (Feng et al., 2000). Five apical dendritic segments, ∼15–20 μm in length and starting ∼150 μm from the neuron’s soma, from each of eight mature Flr males and eight mature WT_FLR_ males, were chosen for quantitative analyses. All scoring of protrusion type was done blinded to the phenotype of the mouse. Flr dendritic shafts had many more irregular, frequently thin protrusions than WT_FLR_ neurons (Fig. 8*A*). We found a significant increase in Flr as compared with WT_FLR_ in filopodia density (Fig. 8*A*, arrows, *p* = 0.02, *B*); no difference in thin spines (Fig. 8*A*, asterisk, *B*) and an increased number of mushroom spines in WT_FLR_ compared with Flr (Fig. 8A, arrowheads, *p* = 0.03, *B*). Together, our data indicates that expression of Flr protein leads to deficits in spine density and maturation. We speculate that these defects are caused by defective transport of synaptic proteins shown by the reduced expression of synaptic proteins in synaptosomal fractions (Fig. 5) and by previous data from our laboratory showing mislocalization of synaptic proteins in Flr mice (Yoshii et al., 2013).

## Discussion

MyoVa has many functions in the body, including within the nervous system. MyoVa is a prominent and versatile motor for delivering cargo close to its target zone (Langford, 2002; Yoshimura et al., 2006). Our results report the consequences of disrupting this motor’s critical role in establishing proper presynaptic to postsynaptic transfer of neurotransmitter-mediated nervous system information. In all neurons with PSDs on dendritic spine tips MyoVa delivers ribonuclear particles for local synthesis of postsynaptic proteins (Yoshimura et al., 2006) and a variety of cargos associated with spine PSD components, such as smooth ER, TrkB, mRNA/protein complexes, the molecule PSD-95 and additional scaffolding. All are crucial for normal glutamate depolarization at spine PSDs and postsynaptic cell activation (Naisbitt et al., 2000; Yoshimura et al., 2006; Wagner et al., 2011; Sui et al., 2015). Consequently, in Flr mice homozygous for a dominant-negative MyoVa mutation, the disruption of the transport of these synaptic components causes severe deficits in synaptic function and behavior.

Many analyses of glutamate PSD structure have established the scaffolding system at glutamate synapses (Sheng and Hoogenraad, 2007). Electrophysiological abnormalities such as disruptions in mGluR LTD and changes in dendritic protrusions in the Flr hippocampus as well the abnormal behaviors of Flr mice indicate that this animal’s truncated MyoVA causes a phenotype similar in some respects to those of mice that have been genetically engineered to have one of their native scaffolding genes altered to mimic mutations found in humans with ASD or afflicted with other neuropsychiatric syndromes (Welch et al., 2007; McFarlane et al., 2008; Peñagarikano et al., 2011; Peça et al., 2011; Peça and Feng, 2012; Ronesi et al., 2012; Sato et al., 2012). These genes include those encoding proteins that are known cargos of the MyoVA transport system, such as SAPAP, Shank, and PSD-95. One example of a shared abnormal behavior is the self-destructive repetitive behavior that causes mice to lose fur or bloody themselves with compulsive grooming of one body region. This behavior is seen with SAPAP and some SHANK mutations and can be eliminated if SAPAP3 is replaced in the striatum (Welch et al., 2007). Mouse models with Shank mutations produce autism-syndrome-like behaviors (Peça et al., 2011; Wang et al., 2011; Wöhr et al., 2011; Sato et al., 2012; Jiang and Ehlers, 2013). Mutations in PSD-95 itself have been seen in some people with ASD (Feyder et al., 2010; Tsai et al., 2012; Cao et al., 2013). In addition, proteins bound at the synapse by these major scaffolds at the PSD, such as multimerized or long Homers 1b or 1c (Ronesi et al., 2012) and neuroligins (Yu et al., 2011; Baudouin et al., 2012; Wöhr and Scattoni, 2013), are also implicated in human autism or autism-like behaviors in mice. The Flr mouse is significant because it is the only *myo5a* rodent mutant where the *myo5a* mutation is brain-specific and the only *myo5a* mutant that survives throughout adulthood, hence able to show abnormal behavior. In addition, the expression pattern of the *flr* mutation’s promoter (*gnb5*) has been mapped in the mouse brain (*Allen Mouse Brain Atlas*), facilitating identification of the contribution of mutation-specific brain regions and to particular behaviors.

The issue of whether the Flr mouse specifically models humans with ASD is complex and impossible to answer at present, since many of the interactions that cause ASD and other neuropsychiatric diseases are unknown. Many mouse mutants with the single mutations seen in autism families show the anxiety, avoidance and disruption of normal vocalizations seen in Flr mice (Figs. 1, 2, 4; Welch et al., 2007; McFarlane et al., 2008; Peça et al., 2011; Peñagarikano et al., 2011). In humans subsequently diagnosed as being on the autism spectrum, early onset seizure with subsequent recovery is often present (Berg et al., 2011; Jeste, 2011), and autistic children are frequently asocial, anxious, show repetitive behaviors and become socially awkward as adults. The Flr mouse shows similar characteristics but its repetitive behavior is elaborate frequently including a full-body grooming sequence. This behavior does not lead to any fur loss since it is not localized similar to what has been seen in other mice models of obsessive compulsive disorder (Welch et al., 2007). Such behavior has not been seen in mouse models carrying single human ASD-associated mutations and might reflect an unknown whole-body somatosensory defect. However, we suggest that such a whole-body sensorial defect is unlikely, since Flr/Flr pairs are able to engage in social interactions with their littermates showing excessive nose-to-anal sniffing and contact (Fig. 2*C*). In addition, the behavior of a Flr mouse in a confined area with a WT mouse is aggressive, as if touching in the head area were sufficiently threatening to a Flr mouse that it needs to push the WT mouse away with its forepaws when approach is near its head (Movies 5, 6). This view is consistent with our findings showing severe anxiety behaviors displayed by Flr mice in the plus maze, and also with the repetitive grooming that occurs whenever Flr mice are in an unfamiliar environment (Fig. 1).

The high expression of *gnb5* in dorsal hippocampus motivated us to test Flr and WT_FLR_ spatial memory using the water maze and the assay for context-dependent freezing. Flr mice failed in both of these tests that are dependent on spatial memory mediated by the in dorsal (CA1) hippocampus (Morris, 1984; Tsien et al., 1996). Conversely, our experiments using a cued fear-conditioning paradigm dependent mainly on the amygdala (McHugh et al., 2004) showed no difference between Flr and WT_FLR_ animals, a finding that correlates with the low expression of *gnb5* and consequently of Flr in this brain area.

Our behavioral data demonstrate significant differences between WT_FLR_ males and FLR males. We did not asses the behavioral performances of females in these tests. Because there were no detectable sex differences in our biochemical, electrophysiological and morphologic findings, we believe that the likelihood of a sex-specific difference in these behavioral tests is low.

The defects observed in the behavior of Flr mice could be explained by the deficient transport of synaptic proteins and endoplasmic reticulum (ER) to the PSD. Examining synaptosomes from Flr mice, we found that the Homer-IP3 receptor complex that binds to Shank and the MAGUKS (PSD95, PSD93, and SAP102) were significantly reduced (Fig. 5). The IP3 receptor is integrated into the smooth ER membrane, enabling the MyoVa motor protein to pull the entire PSD-95 scaffolding complex with the smooth ER into spine synapses (Hammer and Sellers, 2011), thus explaining the deficient localization at the synapse.

Interestingly, SAP102, the earliest appearing MAGUK, was also reduced. SAP102 is not palmitoylated and therefore cannot travel by vesicular transport (El-Husseini et al., 2000). SAP102 is known to traffic to the synapse of young neurons in association with the NMDAR subunit GluN2B (Washbourne et al., 2004) but might not do so in older neurons such as the ones used in this study. GluN2B was not reduced in Flr relative to WT_FLR_ synaptosomes (Fig. 5*D1*,*D2*). For this reason, we suggest that defects in synaptic protein synthesis might explain the decreased SAP102 presence at these older hippocampal synapses since SAP102 synthesis has been observed in isolated CA1 hippocampal neuropil (Cajigas et al., 2012). Defects in local protein synthesis could also account for some decreases in PSD-95, PSD-93, and other synaptic proteins we examined. However, it is interesting to note that as techniques for isolating transcripts in neurons have improved more proteins have been found to be synthesized outside of the neuronal cell body in unexpected dendritic and axon compartments (Cajigas et al., 2012; Holt and Schuman, 2013). The local synthesis of PSD-95 but not of FMRP and β-actin might be defective (Fig. 5) because the granules that transport FMRP and β-actin mRNAs to the synapse for local protein synthesis are transported by kinesin instead of by myosins and hence might not be affected by the Flr mutation (Davidovic et al., 2007).

Our previous work demonstrated that in mice homozygous for the Flr mutation visual cortical neurons have normal NMDAR-dependent LTP but lack NMDAR-dependent LTD (Yoshii et al., 2013). We also showed that in Flr mice many molecules critical to normal glutamate synaptic function are significantly reduced and misplaced throughout Flr dendritic shafts, including AMPAR subunits, dynamin-3 (critical for AMPAR) and other proteins that undergo endocytosis at synapses (Lu et al., 2007; Morimoto-Tomita et al., 2009; Petrini et al., 2009; Selvakumar et al., 2009; Kato et al., 2010). Our current study shows that in the hippocampus mGluR-dependent LTD was also diminished in Flr animals (Fig. 6) and that positive modulators of mGluR did not revert this aspect of the Flr phenotype (Fig. 7). These observations suggest that the reduced transport and localization of synaptic proteins in Flr animals directly affects the induction of LTD, which cannot be recovered by the solely activation of mGluR. The lack of response to CDPPB was expected in Flr mice, because mGluR5-LTD requires protein synthesis that, in turn, requires long Homer in the glutamate scaffold (to maintain the protein synthesizing machinery near the receptor; Tu et al., 1999) and as seen in Figure 5*C*. Homer is significantly depleted in Flr synaptosomes.

Disruption of LTD has been widely associated with ASD in mutant mouse models of TSC1, Pcdh10, or Pten (Bateup et al., 2011; Tsai et al., 2012; Takeuchi et al., 2013). The lack of LTD in these mutant animals severely affect developmental activity-dependent synapse elimination or “pruning.” The absence of LTD and the consequent hyperconnectivity between neurons in transgenic mice carrying human ASD mutations, suggest that depression and elimination of early axon connections might be a highly vulnerable process in the developing brain. Understanding which connections are sensitive to disruption of this important event and how the disruption causes abnormal behavior will be important steps in mechanistically addressing psychiatric disease. We suggest that the Flr mouse could significantly facilitate this endeavor. Indeed, much of the abnormal Flr behavior we describe as well as the differences in hippocampus synaptosome proteins between Flr versus WT_FLR_ mice can be related to the abnormally high level of synaptic function added by LTP but not removed by LTD in this mouse.

The abnormal behaviors of the Flr mutant mouse offer a unique opportunity to explore the importance of LTD and synaptic transport during normal development and behavior. Editing the Flr genome to inactivate at least one copy of the Flr gene should restore both normal MyoVA function and a WT phenotype (Bustos et al., 2015; SFN 390.23/C18). Then conditional knockouts specific to different neuron types could be used to identify the anatomic pathways and brain areas responsible for the abnormal behaviors of Flr mice. Such information might identify orthologous brain pathways disrupted in human disorders that present with abnormal behaviors comparable to those of Flr mice. Subsequently, studies of the *flr* gene could facilitate pharmaceutical or behavioral interventions to mitigate or cure some of the most devastating human neuropsychiatric diseases.

## Synthesis

Reviewing Editor: Lynda Erskine, University of Aberdeen Decisions are customarily a result of the Reviewing Editor and the peer reviewers coming together and discussing their recommendations until a consensus is reached. When revisions are invited, a fact-based synthesis statement explaining their decision and outlining what is needed to prepare a revision will be listed below. The following reviewer(s) agreed to reveal their identity: Linda Dansereau, Chiara Manzini.

Both reviewers agreed that this is an interesting and comprehensive study showing multiple morphological, physiological and behavioural deficits in the Flr mouse likely due to trafficking disruptions through Myo5a. However, we are agreed that, while the data appear strong, the manuscript would benefit from substantial rewriting. In particular, more detail is required on some of the experimental approaches, statistical analyses and experimental outcomes. Care also needs to be taken not to overstate the conclusions of the experiments. Following consultation between the reviewers and myself, below are the points that we are all agreed need to be addressed prior to publication.

Major points:

1. The Methods section requires a major review to ensure all methods and reagents are described completely. For example, there is no description of how the hippocampal homogenates or synaptosomes are prepared.

Limited information is provided on the design and performance of the Morris Water Maze test and what is available is distributed in the methods, results and figure legends. The figure legend indicates that 8 trials per day are performed over 2 days for training. Are all the trials resulting from drops from the same location in the tub, or are they 2 sets of trials with 4 independent drops at different locations? If this is the case, it would be helpful to see the averages of the two daily trials. More information needs to be provided in the methods. A paired t-test across the two days for each genotype may not be the most appropriate statistical method and an ANOVA would be needed.

If there were multiple training days did the Flr mice show any improvement on successive days? The results showing no memory of the location 24 hrs after training suggests there wasn’t.

Other details to review include the description on line 170 referring to a “zoom factor of 2 images”.

2. Swim distance in the Morris Water Maze may not be the most reliable measure unless the authors are absolutely sure that the software picks up the mouse immediately when it is dropped in the tub. In addition, swim distance does not account for floating time. The time between drop and platform should also be provided.

3. The grooming phenotype is interesting, but the authors should add if they observed any fur loss or self-barbering in these mice. This appears to be less severe than obsessive grooming phenotypes observed in other models and it should be stated.

4. In the USV analysis, the author find that the duration of vocalizations is longer, which would argue for increased anxiety in the pups as observed in the adults. Is the frequency of vocalizations changed? Are there differences in the type of syllables produced by the pups? Additional details about these findings would be helpful.

5. A portion of Flr mice have been reported to develop ataxia early in life and recover. Are animals used in this study recovered adults or a mixture? Do the animals who showed ataxia early in life have more severe phenotypes?

6. Line 258: “age matched wild type mice from the Flr genetic background” is unclear. Are these littermates controls? Could the authors specify which controls were used. If the Flr mice have been maintained in homozygosity for several years, it is entirely possible that the controls from a “matched” line are not appropriate and may show mild differences.

7. Only male mice were used in this study. Is this also true for the electrophysiology and biochemistry experiments? Could the authors specify sex of the experimental animals and state why sex as a biological variable was not considered?

8. The description of statistical analysis in the Methods only includes paired Student’s T-test and Sum Rank test, but then Figure 1 indicates the use of an unpaired T-test. The authors should consistently indicate for each figure which statistical test was used for the experiments reported. The possibility that a paired T-test was used throughout the study was worrisome because this test is appropriate only in a very specific subset of cases with repeated measures.

9. The results in Figure 8 do not directly show that “defective transport of synaptic proteins leads to deficits in spine density and maturation” as stated in line 505. Supporting that connection with previous research or showing mis-localisation of synaptic proteins in the neurons with immunostaining would make it clearer within the context of this work.

10. There was a lot of confusion in the Introduction about what kind of mouse model this is. It was defined as a protein truncation controlled by the Gnb5 promoter. In fact, what they are studying is not a truncated protein, but a fusion protein with Gnb5 which was a product of gene fusion and incomplete duplication because of a non-homologous recombination event. Each mouse carries a normal copy of Myo5a and Gnb5 plus a fused gene under some portion of the Gnb5 promoter. The first sentence of the Results has the correct information which should be appropriately reflected in the Introduction because it is very difficult for the reader to figure out whether this is an overexpression model or a protein truncation and how much of the normal Myo5a protein is present.

11. The interpretation of behavioral phenotypes and claims about this being an autism model are overstated. Anxiety, autism and OCD cannot be bundled together as they are clinically and genetically distinct entities. That many ASD models present primarily with anxiety phenotypes is true, but that is an issue with the field at large on how specific mutations may present in the mouse. The authors should be careful in the text to indicate that the mice show anxiety, or social deficits, or learning deficits that are features of specific human disease, and not say that there is autism in mice like in line 272. Also please make sure to use intellectual disability when describing cognitive deficits in humans not mental retardation as in line 478. Overall, these mice appear to have strong anxiety phenotypes, increased grooming, a really fascinating aversion for nose to nose contact (which was very nicely characterized) and hippocampal learning deficits. Whether this is a model of autism it is not clear from the findings.

Minor points:

1. Some proof-reading is required prior to publication

2. In line 261 the authors comment on the assistance by another lab. This would be more appropriate in the acknowledgements section

3. Beta actin is also regulated by local translation at synapses in response to mGluR-dependent LTD from FMRP granules transported by kinesin (eg: Davidovic et al, Human Mol Genetics, 2007). This may explain why neither FMRP nor actin levels are decreased in Flr synaptosomes.
